# Health literacy: the missing link in improving the health of Somali immigrant women in Oslo

**DOI:** 10.1186/s12889-016-3790-6

**Published:** 2016-11-03

**Authors:** Abdi A. Gele, Kjell Sverre Pettersen, Liv Elin Torheim, Bernadette Kumar

**Affiliations:** 1 The Institute of Nursing and Health promotion, Oslo and Akershus University College, P.O. Box 4, St. Olavs plass, 0130 Oslo, Norway; 2 Norwegian Center For Minority Health Research, Oslo University Hospital, Oslo, Norway

## Abstract

**Background:**

Existing studies report a positive association between inadequate health literacy and immigrant’s adverse health outcomes. Despite substantial research on this topic among immigrants, little is known about the level of health literacy among Somali women in Europe, and particularly in Norway.

**Methods:**

A cross sectional study using respondent driven sampling was conducted in Oslo, Norway. A sample of 302 Somali women, 25 years and older, was interviewed using the short version of the European Health Literacy Questionnaire. Data was analysed using logistic regression.

**Results:**

Findings revealed that 71 % of Somali women in Oslo lack the ability to obtain, understand and act upon health information and services, and to make appropriate health decisions. Being unemployed (OR 3.66, CI 1.08–12.3) and socially less integrated (OR 8.17, CI 1.21–54.8) were independent predictors of an inadequate health literacy among Somali women.

**Conclusions:**

Enhanced health literacy will most likely increase the chance to better health outcomes for immigrants, thereby moving towards health equity in the Norwegian society. Therefore, policies and programs are required to focus and improve health literacy of immigrant communities.

## Background

The health literacy term was introduced in the 1970s [1] and it is defined as the “personal characteristics and social resources required for individuals and communities to access, understand, appraise and use information and services to make decisions about health” [2]. According to WHO, health Literacy extends far beyond the narrow concept of health education and individual behavior-oriented communication [2]. It addresses the environmental, political and social factors that determine health, including a wide range of skills, and competencies that people develop over their lifetimes to seek out, comprehend, evaluate, and use health information in order to make informed decisions, reduce health risks, and improve quality of life [3]. These skills include knowledge of risk factors which is often influenced by cultural beliefs, understanding of the local health system, and knowledge of local health information channels such as media and online information channels. Skills and competencies in health literacy are necessary as the contemporary health-care systems has turned out to be more complex, and people are often expected to make their own decisions with regard to health care, disease prevention and health promotion [4]. In light of the recognition that health literacy is an important determinant of health for people, examining immigrants’ level of health literacy is essential to help maximize their future health outcomes. This study investigates the level of health literacy among Somali immigrant women in Oslo area, Norway.

While immigrant communities in Norway have the greatest health burdens, they often have limited access to relevant health information. This could be attributed to the complex and cumbersome ways health information is often presented [5]. Besides individuals need to understand their rights and have the ability to navigate through the health care system as informed consumers of the health services. Only then are they able to influence the local health policy through active advocacy and participation in order to improve their health. Weak health literacy may result in less healthy choices and difficulties in making an informed decision with respect to health promotion activities [6]. Existing literature noted the consequences of low health literacy, including a limited knowledge of preventive and curative services [5, 7], poor physical and mental health [8], as well as a higher frequency of hospital admissions with increased morbidity and mortality [9].

More people have been forcibly imposed to migrate outside their natural habitat, while globalization has also increased the possibility of people to voluntarily migrate to a country other than the one in which they were born. After arrival to the new country, immigrants go through a period of uncertainty. The host country’s capability to an early integration of immigrants into a tailored health system may determine the long-term health status of immigrant populations. Immigrant populations are often vulnerable to serious health disparities, with many immigrants experiencing significantly worse health outcomes, such as higher rates of diabetes and other non-communicable diseases, than other segments of society [10, 11]. Prior studies have found that half of the adult refugees had at least one chronic non-communicable disease (NCD), while 10 % had three or more NCDs [12], with half being overweight or obese (54.6 %) [12]. Similarly, the prevalence of hypertension, diabetes, obesity, metabolic syndrome and other NCDs were found to be higher among immigrants than in the host populations [13–15]. Although migrants are often initially healthier than non-migrant populations, changing environments and the consequent undesirable lifestyle, which is largely precipitated by a low health literacy [16], may expose them to chronic NCDs [13]. On arrival, immigrants have less knowledge of the health system, and therefore limited access to preventative health-care services and information, both of which may negatively affect their health over time [17]. Previous studies investigated what happens to ‘healthy migrant effect’ and found that immigrants use fewer resources for health than the mainstream population, and the used resources are for treatment rather than for prevention [18]. Accordingly, people with lower literacy skills are 1.5 to 3 times more likely to experience negative health outcomes and difficulties in managing chronic diseases [19], in addition to the underutilization of preventive health-care services [20, 21]. For example, Somali refugee women were reported to focus on issues of immediate survival, with many of them having no reference for the notion of prevention and the long-term management of chronic diseases [22].

Norway’s immigrant population (immigrants and Norwegians born to immigrant parents) rose from 183,000 persons (4.3 %) in 1992 to 759,000 persons (14.9 %) in 2014 [23]. There are immigrants in all Norwegian municipalities, with highest percentages in Oslo, where immigrants and their descendants comprise 32.5 % of the population. In 2012, there were approximately 200,000 refugees, constituting approximately 4 % of the Norwegian population and roughly 30 % of total immigrants in the country. The majority of refugees are from Somalia [24], and a low health literacy can be expected from this group as they navigate a new country, language and culture [25]. Somalis arrive in Norway with different health-care experiences and knowledge of health issues. They also experience changes in family composition, and traditional ways of sharing health information may be fragmented, with such experiences having a profound impact on the way Somali refugees engage with health information, health-care services and preventive health activities. For maintaining their own health and the health of their communities, immigrants rely heavily on the health information available to them. However, health literacy is not only a personal attribute or a one-way process that depends upon an individual’s ability to comprehend the information, but it also involves health-care providers’ competencies in multicultural health provision, the “legibility” of the health-care system for diverse groups, and appropriate policies and programs to achieve effective communication [17].

Like other countries in Western Europe, the burden of diseases in Norway is more clustered toward immigrant communities [26]. Limited access to health information regarding preventive health and the poor utilization of preventive health services was observed among Somali women in Norway [5]. A prior study in the United States found that Somali women are less likely to be screened for breast, cervical and colorectal cancer than other immigrant women, whereas the uptake of colorectal cancer screening is associated with years of residency in the host country [27]. A current study in Sweden reported a 60 % rate of inadequate health literacy among refugees, and factors such as being born in Somalia were associated with an increased risk of having an inadequate health literacy [25]. Health literacy is an important predictor in the utilization of preventative health measures among immigrants [28, 29], with the literature suggesting that any future efforts to improve equity in disease prevention may require an improved health literacy of immigrants [27]. Nonetheless, despite Somali women in Norway being reported to have higher rates of overweight and obesity [7], a high risk of diabetes that increases with the duration of residence in Norway and a lower utilization of preventive health services, the level of health literacy among this group has never been studied in Norway.

## Methods

### Recruitment of participants

Most of the prior quantitative studies on African immigrants have used a non-probability sampling design [7]. Because the sample frame of this immigrant community is rarely available or accessible, conventional methods of selecting a random sample for the cross-sectional surveys could not be applied. Moreover, the existing non-probability sampling methods for immigrants, i.e. snowball sampling, introduce well-documented sampling biases, whereby no statistical inference from the sample to the larger target population can be made with any accuracy [30]. Still, as postulated by the literature of the “small world”, this approach could potentially reach all members in the target population in only six waves [31], so total coverage is very likely with a resultant statistically invalid sample of broader coverage. Consequently, we used a chain referral sampling technique with respondent-driven sampling (RDS), which helped to overcome this dilemma by combining the wide coverage of network-based methods and the statistical validity of standard probability sampling methods [32]. RDS is a chain referral method, with the major difference between snowball sampling and RDS being that seeds recruit their peers, rather than identifying them to an investigator using a uniquely coded coupon [33]. This type of peer-to-peer recruitment removes any selection bias that may be created by the survey staff, while the use of an equal number of coupons for the recruitment minimizes biases associated with the over-representation of those participants with large networks [33]. Although we used the RDS method in the recruitment of participants, we treated data as ordinary snowball sampling during the analysis because we had no intention to calculate population estimates and generalize data to the population as assumed by the RDS method.

### Data collection

The eligibility criteria for the study included being a Somali female, permanently and legally residing in the Oslo and Akershus regions of Norway, aged ≥25 and being willing to provide informed consent. Initially, a formative study involving five Somali women was conducted with the aim of understanding the social network structure of Somali women, the average peer that each recruiter can recruit and the recruitment incentives necessary to motivate Somali women to participate in the study. A respondent-driven sampling design suggests that seeds should be persons who are socially connected and motivated to recruit others [32]. For this reason, three eligible seeds, comprising socially well-connected women, were purposely selected based on a diversity of location, years of stay in Norway and age. After providing informed consent, the seeds underwent an interview, were educated on how to refer other eligible Somali women and were given two uniquely coded coupons to help refer their peers in their social network. The reason for only using two coupons was to elongate the recruitment waves so that the diversion of subsequent waves from the initial seeds was increased. Each seed proceeded to recruit two persons from their network, which became the first wave, with the first-wave participants further recruiting their peers, which then became the second wave. Each participant was compensated with 100 Norwegian kroner (NOK) (12$) for participating in the study, and received an additional 100 NOK for each recruited peer who met the eligibility criteria and participated in the study. The chain referral process continued until we obtained the desired sample size of 302, which was calculated using the formula necessary to determine the sample size required for estimating the sample proportions. The study was ethically approved by the Norwegian center for research data (NSD).

### Measuring health literacy

The study used a short version of the European health literacy questionnaire (HLS-EU-Q16), which contains 16 statements and was developed by the HLS-EU Consortium for measuring the health literacy of general populations and not of specific patient groups [34]. It does not follow a narrow clinical or medical focus, but instead captures a broad public health perspective, with wider expert consultations making the questionnaire applicable to different cultures [35]. The statements in the questionnaire cover three different health literacy domains, including Health Care, Disease Prevention and Health Promotion. Each respondent was asked to give her opinion on a 5-point Likert scale (very easy, fairly easy, fairly difficult, very difficult, I don’t know). Each statement was given a numeric value with statements rated by respondents as being “very difficult” as having a numeric value of “1”, and statements being rated as “very easy” as having a numeric value of “4”. Statements that were answered as “Don’t know” were given a 0, but later treated as missing data in the analysis. For each respondent, the average value of all statements was worked out and then converted to a metric score in order to obtain the relevant HL index using the following formula:

$$ \mathrm{Index}=\left(\mathrm{Mean}-1\right)*\left(50/3\right) $$

Where:

Index is the specific index calculated

Mean is the mean of all participating items for each individual

1 = the minimal possible value of the mean (leads to a minimum value of the index of 0)

3 = the range of the mean

50 = the chosen maximum value of the new metric.

The metric scores for HL indices ranged between “0” and “50” with “0” being the lowest value and “50” the maximum. The same method was applied when working out indices to determine the HL with respect to health care (HC), disease prevention (DP) and health promotion (HP). Respondents having a score of 25 or less were categorized as having an “inadequate” HL; persons scoring between 25.1 and 33 were deemed to have a “problematic” HL, whereas individuals with a score from 33.1 to 42 were assumed to have a “sufficient” HL. Persons obtaining a score over 42 to 50 were deemed to have an “excellent” level of HL. In a logistic regression analysis, the “inadequate” and “problematic” categories were collapsed together and coded as 1, whereas the “sufficient” and “excellent” categories were collapsed together and coded as 2. The former category was labelled as a “limited health literacy”, while the latter was labeled as an “adequate health literacy”.

The general health literacy (GHL) was determined by the combination of answers of the 16 statements mentioned in Table 1. Health Literacy (HL) with respect to Health Care (HC), has been determined through seven statements [1–7] and Disease Prevention (DP) through five statements [8–12], whereas Health Promotion (HP) was determined through four statements [13–16]. As shown in Table 2, the Cronbach’s alpha in overall health was 0.91, in health communication 0.87, in disease prevention 0.79 and in health promotion (0.70), which indicates a high level and satisfactory internal consistency for our scale with this specific sample.

**Table 1:** Percentage distribution of responses for HL statements

No.	Questions	Very easy	Easy	Difficult	Very difficult	Don’t know
1	Find information on treatment of illnesses that concern you	19.5	54	15.2	5.3	5
2	Find out where to get professional help when you are ill	24.8	58.9	10.6	4.3	0.3
3	Understand what your doctor tells you	20.2	49.7	21.5	6.6	1
4	Understand your doctor or pharmacist’s instructions on how to take a prescribed medicine	24.5	56	12.3	5.6	1
5	Judge when you may need to get a second opinion from another doctor	13.6	39.1	33.4	10.3	2
6	Use information the doctor gives you to make decisions about your illness	15.9	58.3	17.2	5	1.7
7	Follow instructions from your doctor or pharmacist	19.5	61.9	12.6	4	.3
8	Find information on how to manage mental health problems like stress and depression	9.6	35.1	26.2	24.2	4.3
9	Understand health warnings about behavior such as smoking, low physical activity and drinking too much	15.9	42.4	26.2	10.3	3.6
10	Understand why you need health screenings	17.5	59.3	12.9	3	6.3
11	Judge if the information on health risks in the media is reliable	8.6	36.1	32.8	16.9	2.6
12	Decide how you can protect yourself from illness based on information in the media	10.9	33.8	32.8	19.9	1.7
13	Find out about activities that are good for your mental well-being	12.9	41.1	32.5	11.9	1.3
14	Understand advice on health from family members or friends	43.7	41.4	9.3	3.3	1.3
15	Understand information in the media on how to become healthier	10.3	35.1	30.8	22.8	.7
16	Judge which everyday behavior is related to your health	19.2	42.1	24.2	13.6	.7

**Table 2:** The indices of general health literacy, health care, disease prevention and health promotion

	Number	Inadequate (%)	Problematic (%)	Sufficient (%)	Excellent (%)
GHL	213	29.6	41.8	17.8	10.8
HC	266	18.0	31.6	35.7	14.7
DP	247	44.1	29.1	17.4	9.3
HP	290	36.9	20.7	34.8	7.6

**Cronbach’s alpha**

**Table:** 

**Health Literacy Indices**	**Cronbach’s alpha**
**General Health Literacy**	0.91
**Health care**	0.87
**Disease Prevention**	0.79
**Health Promotion**	0.70

### Statistical analysis

Data was analyzed using the SPSS version 20.0 statistics program. We performed a chi-square test for the analyses of categorical variables and a t-test for continuous variables. A binary logistic regression was used to investigate the association between general health literacy and the independent variables, and a crude model was run to investigate the association of each independent variable and the general health literacy. Afterwards, an adjusted model was run to determine variables associated with a low health literacy when the effect of other variables are adjusted (Table 5). The association was assessed by using a 95 % confidence interval (CI) and odds ratio (OR), with the level of significance being determined at a *P*-value <0.05.

## Results

A total of 302 Somali women, age ≥25, were interviewed from September to November 2014. The mean age of the study population was 36.13 ± 8.0 SD, whereas the mean years of residence in Norway were 9.9 ± 6.4 SD. The majority of the study population (70 %) had only a primary- or no education, with only 6 % having a college or university education level. Almost 45 % of the study participants had lived in Norway ≥11 years, while 31 and 24 % had lived in Norway for ≤5 years and 6–10 years, respectively. Regarding health literacy, it is evident that for all of the 16 statements identified in Table 1, the majority of respondents felt that the issue being asked about was “easy” or “difficult” to handle.

As indicated in Table 2, 29.6 and 41.8 % of study participants had inadequate and problematic general health literacy, respectively. Only 28 % of the study sample had either sufficient or excellent general health literacy. However, the lowest health literacy was found in disease prevention indices, in which 44 % of study participants had inadequate health literacy in disease prevention. One in every two participants had sufficient or excellent health literacy in the health communication index (50 %).

As indicated in Table 3, the mean GHL in this study was 29.7. The lowest mean was found in disease prevention (26) indices (Table 3). The presentation of the mean HL is crucial for comparing the study with other HL studies.

**Table 3:** Health Literacy index for General Health Literacy, Health Care, Disease Prevention and Health Promotion

	GHL index	HC index	DP index	HP index
Mean	29.7	31.7	26	28.7
SD	9.2	9.9	10.6	10.4

Table 4 illustrates the percentage distribution of HL levels by different sociodemographic variables. The distribution reflects the percentage distribution for general health literacy. No significant difference in health literacy was found among age groups, but general health literacy levels increased with increasing levels of education. Persons with a tertiary education and persons with a secondary level of education had better general health literacy levels than persons with lower levels of educational attainment (*p* = 0.001). When analyzing the information by the employment status of participants, significant differences were found between those who were employed and those unemployed (*P* = 0.000). Similar differences were found between those who had lived in Norway for a longer period (11 years and above) and those who were newly arrived (*P* = 0.030), as well as those who were highly acculturated and those who were not (*P* = 0.000).

**Table 4:** Group differences in general health literacy

Health literacy (%)		Inadequate	Problematic	Sufficient	Excellent	*P* value
Age	25–35	27.2	39.8	22.3	10.7	0.252*
	36–45	27.2	48.1	14.8	9.9	
	>45	45.8	29.2	8.3	16.7	
Education	No school	45.8	45.8	8.3	0	0.001*
	Primary	29.2	40.6	19.8	10.4	
	Secondary	22.4	44.9	20.4	22.2	
	University	12.5	25	25	37.5	
Employment	Employed	16.7	30.6	19.4	33.3	0.000*
	Job training	28.3	40.4	24	6.7	
	Unemployed	34.3	50.7	9	6	
Years in Norway	≤5	35.8	50.9	11.3	1.9	0.030*
	6–10	32.0	42	20	6	
	11–15	22.0	39	22	16.9	
	>15	28.6	34.3	14.3	22.9	
Acculturation	High	17.6	25.7	31.4	23.5	0.000*
	Moderate	23.5	51.9	18.5	6.2	
	Low	47.1	47.1	4.4	1.5	

p* = likelihood ratio

As indicated in Table 5, age was not associated with health literacy. However, those who had a primary or lower level of education were more likely to have a limited health literacy compared to those with a secondary or tertiary level of education (Model 2). Although being newly arrived in Norway and having a primary or lower level of education has exhibited a significant association with health literacy, they lost significance after the acculturation variable was added into the model (model3). A multivariate logistic regression analysis showed that unemployment (OR 3.66, CI 1.08–12.3) and poor acculturation (OR 8.17, CI 1.21–54.8) were independent predictors for a limited health literacy among Somali women in Norway (Table 5).

**Table 5:** Factors associated with a low health literacy

Indicators	Crude OR(CI)	Model 1 OR(CI)	Model 2 OR(CI)	Model 3 OR(CI)
Age				
> 45	1.00	1.00	1.00	1.00
36–45	1.o1 (0.52–1.95)	1.94 (0.47–7.92)	3.94 (0.82–18.9)	4.20 (0.78–22.5)
25–35	0.71 (0.26–1.93)	1.76 (0.45–6.07)	2.04 (0.49–8.39)	1.69 (0.38–7.4)
Education				
University\secondary	1.00	1.00	1.00	1.00
Primary	7.71 (2.42–23.2)*	5.01 (1.39–18.1)*	4.49 (1.02–18.9)*	2.04 (0.40–10.2)
No to school	4.76 (1.56–14.4)*	4.62 (1.48–14.4)*	3.94 (1.04–14.9)*	2.50 (0.61–10.2)
Employment				
Employed	1.00	1.00	1.00	1.00
Job training	6.37 (2.49–16.2)*	4.79 (1.64–13.9)*	3.73 (1.15–12.10)*	2.95 (0.72–12.0)
Unemployed	2.53 (1.14–5.58)*	3.09 (1.25–7.6)*	4.42 (1.58–12.39)*	3.66 (1.08–12.3)*
Years in Norway				
> 15	1.00		1.00	1.00
11–15	2.30 (0.83–6.37)		2.10 (0.59–7.40)	1.20 (0.25–5.67)
6–10	4.19 (1.62–10.8)*		7.07 (1.99–25.1)*	4.48 (0.91–21.9)
≤ 5	3.88 (1.35–11.1)*		4.27 (0.96–18.9)	2.30 (0.34–15.3)
Acculturation				
High	1.00			1.00
Moderate	19.4 (6.16–61.5)*			8.17 (1.21–54.8)*
Low	5.24 (1.69–16.2)*			5.28 (0.94–29.7)

*Significant at *P* < 0.05

Module 1 = crude for descriptive purpose. Module 1 involves age, education and employment. Module 2 = Module 1 + years of residence in Norway. Module 3 = Module 2 + acculturation

## Discussion

The present study shows that 71 % of Somali refugee women in Oslo lack the capacity to obtain, understand and act upon health information and services, and to make appropriate health decisions. The proportion of Somali refugee women in Oslo who lack health literacy skills is significantly higher than the proportion reported (47.6 %) from eight European countries [36] and Germany [37]. This finding indicates that refugees coming to Norway tend to be unfamiliar with the Norwegian health-care system, with health professionals in Norway reporting difficulties in communicating effectively with immigrants about risk-taking behaviors [38, 39]. Refugees are often challenged to make healthy lifestyle choices and manage their health through complex environments and health-care systems, but are not being prepared or adequately supported in addressing these tasks [9]. Therefore, they may not seek care because they may not know where to go or do not think they are sick enough to require the effort associated with seeking care, which may ultimately create health inequality [40]. Norway is a country with a universal health-care system financed primarily through income taxes, and for many years the Norwegian health-care system has sought to reduce health inequalities through prevention and health promotion directed towards immigrants and other vulnerable groups. Nonetheless, there is a cause for concern because a very high proportion of refugee women with low health literacy skills may widen the health inequality gap in Norway. Multidisciplinary work to enhance health literacy and an awareness about health and healthy lifestyles will permit immigrants to develop their potential and more fully enjoy their lives in Norway.

The WHO pointed out that educational resources and information programs only partially reach immigrants, often because of economic, cultural and social barriers [2]. In addition, the e-health information and written materials, which are among the most common means of delivering health information to the public in Norway, do not reach those with language and literacy limitations. The reason being because 80 % of the world’s population live in oral and visual cultures, i.e. cultures that learn through listening and watching, and not through reading or writing [41], with Somali refugee women in Norway falling into that category. Somali refugee women may not participate in health promotion initiatives, and the problems of adapting to a new health culture are linked to both a lack of information about the new health care available and their subsequent experience with that health-care system [5]. Somali women’s lower health literacy skills can have a significant impact on information exchange about health and help-seeking for immigrant families because women often play a central caregiving role in their families and other networks within the community. Therefore, health planners should pay more attention to the health literacy of refugee women because these are areas in which refugees are especially disadvantaged, and it may have wide implications on the health outcome of immigrant families. Attention must be directed to recently arrived immigrants and those with lower levels of education and low Norwegian proficiency. With regard to Somali refugees, given their unique circumstances, special adaptations are required to develop policies and programs appropriate to their needs.

One of the concerns is that the lowest level of health literacy was seen in disease prevention indices, which may reflect the complexity of the health information and preventive health measures available to immigrants. A prior study noted that preventive health services and communications are often not tailored to the needs of Somali immigrant women [5]. Effective disease prevention requires health literacy skills to successfully make informed decisions and healthy behavioral changes [42]. Barriers to health literacy, such as a lack of meaningful information about health issues or how to access preventive services, may contribute to the deterioration in health status of immigrants over time [43]. The difficulties immigrant people face as they attempt to use the Norwegian health-care system may result in inequities in health status and access to care and the outcomes that persist. According to Nutbeam, tailored health literacy interventions may lead to an improved health literacy, which in-turn leads to improved healthy lifestyles and ultimately to a reduced morbidity or an improved health-related quality of life [4].

The study found discrepancies in health literacy between participants who are employed and those who are not, as well as those who are acculturated and those who are not, with employment and social integration being the predictors of an adequate health literacy among the study participants. People with limited financial and social resources are more likely to have a limited health literacy, and the people most at risk of a low health literacy are also known to have the poorest health outcomes [44]. A previous study in Norway associated immigrants’ high level of integration and employment with a good mental health status [45]. Similarly, prior studies demonstrated a clear association between refugees’ unemployment and worse health outcomes [46, 47]. Others pointed out that immigrants who have jobs and skills are healthier than those who have not, and those who are socially integrated are healthier than those who do not [48]. A recent study found that health literacy is often distributed through social network [49]. Therefore, social capital variables such as employment and integration are vital for immigrants’ social network, as they create channels for information and knowledge that may increase immigrant’s health literacy [48]. The result of current study challenges health providers and policymakers for service provision reform to ensure equity since the current system clearly favors individuals with higher socioeconomic backgrounds, such as those who are employed and those who are well acculturated.

The study limitations include its cross-sectional design, which limits drawing causal conclusions. Another limitation is the self-reported survey, which is prone to a number of biases. In addition, the ability and motivation to fill out a health survey can be viewed as a health literacy competency in itself; thus, this study may be over-represented by those with a certain level of health literacy skills. In order to tackle this potential weakness, we recruited the study sample through RDS recruitment method, which has minimized the sampling bias. Therefore, we believe that the study did not underestimate or overestimate the health literacy challenges of the Somali immigrant women. One of the strengths of this study is its presentation of the health literacy of Somali women, who are among the most vulnerable groups in Norway. Several studies presented aggregated information on immigrants, which may mask true the health disparities that each subgroup faces, while findings from this study enable us to give primary attention to the most vulnerable groups.

## Conclusions

While health literacy is seen as a right and issue of equity and citizenship [4], the reported lack of health literacy skills among Somali refugee women in Oslo demonstrates the urgency of a renewed focus on health literacy and in health-care planning to tailor the system to refugee women’s needs. Health literacy requires empowerment of immigrant communities to act upon the factors that affect their health through providing relevant knowledge, skills and competencies. The efforts to improve health literacy must be multi-sectoral involving the combined and coordinated efforts of all major stakeholders [3] including health sector, immigrant communities, education sector, civil society, research institutions and other important stakeholders. Most importantly, policies and programs are required to reduce the individual and systemic barriers to health literacy among immigrant communities. Moreover, research is required that supports policy and practice development, and better understanding of the impact of health literacy on health and well-being of the immigrant population and in particular, the role of health literacy in health disparities in Norway.

## Abbreviations

DP: Disease prevention
GHL: General health literacy
HC: Health care
HL: Health literacy
HP: Health promotion
NCDs: Non-communicable diseases
OR: Odd ration
RDS: Respondent driven sampling
SD: Standard deviation
WHO: World Health Organization
